# American Medical Association

**Published:** 1851-07

**Authors:** 


ARTICLE II.

### AMERICAN MEDICAL ASSOCIATION.

The recent meeting of this Association at Charleston, S. C., we are glad to learn, was a very pleasant and profitable one. Although not as large in point of numbers in attendance as either of the two preceding meetings, the representation from points remote from the place of its meeting, was quite as great as at either.

As the profession generally take an interest in the Association, we have devoted a considerable space, in the present number of the Journal, to its proceedings.

The most noteworthy feature of its proceedings, is the alteration of the Constitution, by which the former standing committees have been abolished. A long list of Special committees, numbering twenty-seven in all, have been appointed in stead of the old arrangement.

Whether this is reform or a retrograde movement, remains to be seen. If to get rid of spending time, in hearing the reports read, was a main object in doing away with the old committees, the matter is not likely to be mended; for twenty-seven reports will probably take up much more time than seven. If the old committees, in making up their synopsis of the subjects committed to them, were in the way of original investigations, it may be well to have them abolished. But certainly a collection of the "more important improvements" into form for permanent preservation, was an object worthy of the association, and we feel quite confident that the loss of it will hereafter be seriously regretted.

The offering five fifty dollar prizes for volunteer essays, we believe will be a very valuable means of calling into active operation the investigating talents of the profession. Not that the money will pay for very able papers, but the distinction to be acquired by taking prizes, will be very much sought after, from which much good may result.

The site of the next meeting will be at the head quarters of the Old Dominion, within a few hours ride of Washington City, where Congress will be in session, and we may expect quite a large delegation to it from distant regions of the country, in consequence of the additional inducements to go that may be found in a visit to the Federal City,

